# Bone regeneration property of tooth-derived bone substitute prepared chairside for periodontal bone defects: an experimental study

**DOI:** 10.1186/s12903-023-03582-y

**Published:** 2023-11-14

**Authors:** Rui Zhang, Nisarat Ruangsawasdi, Piyapanna Pumpaluk, Quan Yuan, Yi Peng, Dutmanee Seriwatanachai

**Affiliations:** 1 Department of Oral Biology, Faculty of Dentistry, Mahidol University, Bangkok, 10400 Thailand; 2 Department of Periodontics, School and Hospital of Stomatology, Kunming Medical University, Kunming, 650106 China; 3 Yunnan Key Laboratory of Stomatology, Kunming, 650106 China; 4 Department of Pharmacology, Faculty of Dentistry, Mahidol University, Bangkok, 10400 Thailand; 5 Department of Advanced General Dentistry, Faculty of Dentistry, Mahidol University, Bangkok, 10400 Thailand; 6 State Key Laboratory of Oral Diseases & National Clinical Research Center for Oral Diseases, West China Hospital of Stomatology, Sichuan University, Chengdu, 610041 China

**Keywords:** Bone regeneration, Bone graft, Tooth particle, Periodontitis, Dental material

## Abstract

**Background:**

Periodontitis often leads to progressive destruction and loss of alveolar bone, the reconstruction of which remains difficult in periodontal therapy. As a novel bone graft material, tooth-derived bone substitute (TDBS) processed from extracted teeth has been previously reported about its osteoconductivity and promising results in bone regeneration. This study was to investigate the biological effects and bone regeneration properties of TDBS in vitro and in vivo using rat periodontal bone defect model.

**Methods:**

Three groups of materials were used in the experiments: TDBS, TDBS treated with ethylene diamine tetraacetic acid (EDTA) (TDBS-E), and allogeneic bone materials. Calcium (Ca) and phosphate (P) ion dissolutions were quantified by spectrophotometer for seven days. The releases of bone morphogenetic protein-2 (BMP-2) and transforming growth factor-β1 (TGF-β1) were identified by enzyme-linked immunosorbent assay (ELISA). Human osteoblast proliferation, migration, and differentiation were detected by 3-(4,5-dimethylthiazol-2-yl)-2,5-diphenyltetrazolium bromide (MTT) assay, cell counting, alkaline phosphatase activity (ALP), and alizarin red staining (ARS), respectively. Furthermore, the osteogenic effects of TDBS on periodontal furcation bone defects were evaluated at eight weeks postoperatively using micro-computed tomography (Micro-CT) and histological analysis.

**Results:**

The dissolution of both Ca and P ions in TDBS increased over time. The BMP-2 released from TDBS was significantly higher than that from TDBS-E and allografts, while the TGF-β1 release from TDBS and TDBS-E groups was higher than that in the allografts. The TDBS-E group could induce the highest level of osteoblast proliferation compared to other groups. Cell migration with allografts co-culture was significantly induced compared to the blank control. However, all groups demonstrated similar positive effects on osteoblast differentiation. Furthermore, in the periodontal model, all materials could effectively enhance bone regeneration in the furcation defect.

**Conclusions:**

The TDBS prepared chairside as an autogenous bone graft, demonstrating osteoinductivity, which enhances the osteogenic biological characteristics. Therefore, TDBS is suggested as an economical and biocompatible material for periodontal bone regeneration.

**Supplementary Information:**

The online version contains supplementary material available at 10.1186/s12903-023-03582-y.

## Background

Periodontitis, a chronic immune-inflammatory disease of the periodontium, is a highly prevalent condition [1] that causes the loss of supporting tissues in the periodontium, leading to the progressive destruction of alveolar bone and tooth loss that can impact mastication and aesthetics. Despite many advances in periodontal therapy, periodontal tissue reattachment and bone regeneration remain challenging for dentists.

In dentistry, bone graft materials have been clinically used to enhance bone formation and regeneration in socket preservation, ridge augmentation, sinus lifting, and regeneration of periodontal bone defects [2]. Three types of bone formation in grafts have been characterized: osteogenesis, osteoinduction, and osteoconduction [3]. While autogenous bone grafts are considered the golden standard because they possess all three properties and have no potential complications of histocompatibility, their use is limited due to the need for bone harvesting, rapid absorption, and potential higher morbidity [4]. Conversely, most other types of bone grafts, such as xenografts and alloplastic grafts, only exhibit osteoconductive properties as matrices and structural scaffolds for anchorage-dependent osteoblast attachment and proliferation [5]. However, allografts and xenografts may cause immunologic, infectious, and inappropriate fibrosis repair [6].

Extracted teeth, which were previously considered medical waste, are now being repurposed as alternative bone substitutes due to their biochemical similarity to the bone. Both the jawbone, alveolar bone, and teeth originate from neural crest cells and contain many proteins, including bone morphogenetic proteins (BMPs), insulin-like growth factor-II, and transforming growth factor-β (TGF-β), which promote bone remodeling [7, 8]. In terms of composition, teeth and bone share similar proportions of inorganic and organic components. Dentin, which makes up over 85% of the tooth, is composed of 65–70% inorganic components and 30–35% organic components. Similarly, cementum shares a comparable composition with the alveolar bone, with approximately 45–50% inorganic and 50–55% organic components [9]. Non-collagenous proteins like osteopontin, osterix, osteocalcin, and runt-related transcription factor 2 (RUNX2) have also been identified in dentin, making it similar to the bone and a potentially effective alternative to other bone grafting materials [10].

Numerous studies have demonstrated the biocompatibility and osteoinductive properties of the demineralized dentin matrix (DDM), which has proven to be effective in promoting bone formation during dental surgeries [11–14]. However, the process of tooth demineralization is time-consuming and impractical in the clinic setting, thereby restricting the use of freshly demineralized teeth as graft material. A novel bone substitute derived from teeth, known as the tooth-derived bone substitute (TDBS), has shown great results in bone regeneration based on several clinical case reports and animal studies [15–20]. TDBS is obtained from undemineralized tooth particles using a dentine grinder that is easily cleaned and disinfected within half an hour.

Moreover, the physicochemical analysis of TDBS revealed that its surface structure and physicochemical properties bear a resemblance to those of bone and other bone graft materials [21, 22]. Therefore, to further investigate the osteoinductive and biological effects of TDBS, this study conducted both in vitro and animal models of experiments focused on periodontal bone defects. We hypothesized that TDBS could provide biological osteogenic properties when co-cultured with human fetal osteoblasts (hFOB) and promote new bone formation. Therefore, TDBS, when used as a grafting material, could effectively achieve bone regeneration in the rat model of periodontal furcation defect. The results obtained will provide evidence and suggest a promising pathway for the future use of TDBS as a potential regenerative periodontal treatment in clinical settings.

### Design of the study

This study was designed to investigate both in vitro and in vivo experiments. The flowchart was created to illustrate the sequence of the entire study, as shown in Supplemental Fig. 1. In brief, the in vitro study aimed to examine and compare the biological effects of TDBS, TDBS treated with ethylene diamine tetraacetic acid (EDTA) (TDBS-E), and allograft (DO BONE™) on hFOB. Subsequently, the in vivo study aimed to evaluate new bone formation after grafting surgery using TDBS, TDBS-E, and allograft (BIO-GENE™) in rat periodontal furcation defects.

## Methods

### Teeth collection

The protocols involving human samples of this study were carried out in accordance with the ethical approval by the Faculty of Dentistry/Faculty of Pharmacy, Mahidol University Institutional Review Board (MU-DT/PY-IRB 2020/029.1007) under the principles of the Declaration of Helsinki. Extracted teeth were collected from the Faculty of Dentistry, Mahidol University, Thailand, and the School and Hospital of Stomatology, Kunming Medical University, China, after obtaining ethical committee approval (KYKQ2020MEC007). The informed consent was obtained from all subjects and/or their legal guardian(s). The table described the materials used in the study was shown in the supplemental Table 1 (table S1).

### Inclusion and exclusion criteria

Patients aged 13–50 years who were non-smokers and non-alcoholics and had teeth extracted due to impaction or for orthodontic purposes were included in this study. Patients with infectious diseases, such as hepatitis B, hepatitis C, or human immunodeficiency virus that could be transmitted through their teeth were excluded. Teeth with extensive caries or previously undergone dental treatments were also excluded, including fillings, root canal treatment, restorations, or prosthetic crowns. Furthermore, tooth malformation or congenital dental anomalies, such as fluorosis, enamel hypoplasia, amelogenesis imperfecta, dentinogenesis imperfecta, and dentin dysplasia, were ruled out as well. The table described the inclusion and exclusion criteria of teeth collection was shown in the supplemental Table 2 (table S2).

### Preparation of TDBS and TDBS treated with EDTA (TDBS-E)

After tooth extraction, small fillings, caries, discolored dentin, dental calculus, and residual periodontal ligament were immediately removed by high-speed tungsten burs. The Smart Dentin Grinder™ (SDG) (Kometa Bio, USA) was used to grind the extracted teeth and sift particles into a specific size. TDBS was prepared according to the manufacturer’s instructions. Briefly, the completely cleaned tooth, including the crown and root, was air-dried and placed in a sterile room. The particulates, sizes between 300 and 1200 μm, that passed through the sieve were collected in the upper drawer. These particles were submerged in a basic alcohol cleaner containing 0.5 M of NaOH and 30% alcohol (v/v) for 10 min to degrease and dissolve all organic debris, bacteria, and toxins [15]. The TDBS-E group was treated with 10% EDTA for 5 min before rinsing. After the chemical solutions were poured off, the particles were washed three times with sterile phosphate-buffered saline for 3 min each time. The tooth particles remained wet throughout the preparation process, which took approximately 15–20 min.

### Allografts

DO BONE™ (Biotem, Korea) is a cancellous and cortical bone commercial product used in vitro studies. BIO-GENE™ (Datsing, China) is a mixture of cancellous and cortical bone used in vivo studies. The disinfection and cleaning procedures were performed the same as those of TDBS in this experiment.

### Part I: in vitro study

#### Calcium and phosphate ions dissolution test

The 0.25 g of TDBS sample was added to 5 mL of deionized water (DI water) and kept in an incubator (INB-500, Memmert, Germany) at 37℃. On day 1, the DI water containing 0.25 g of TDBS was centrifuged (Universal 320 R, Hettich, Germany) at 1000 rpm for 5 min to obtain 4 mL of supernatant and supplemented with a fresh 4 mL of DI water. The same procedure was repeated on days 3, 5, and 7, and the sample was stored at room temperature. Subsequently, the amounts of Ca and P ions were measured in the supernatants obtained on days 1, 3, 5, and 7. The absorbance was determined by the spectrophotometer (Bio-Tek, USA) at 570 nm for Ca and 810 nm for P, respectively. All experiments were done in triplicate.

### Growth factors quantification with enzyme-linked immunosorbent assay (ELISA)

One gram of each material from TDBS, TDBS-E, and DO BONE was transferred into a 24-well plate with 1 mL of serum-free Dulbecco’s Modified Eagle Medium/Nutrient Mixture F-12 (DMEM/F-12) (Hyclone, USA) and incubated the same condition with the other in vitro studies for monitoring the released growth factors. A serum-free DMEM/F-12 medium without material was used as a blank control. 900 µl of supernatant medium from each well was harvested at 1, 3, 5, 7, and 10 days. Meanwhile, 900 µl of new serum-free DMEM/F-12 medium was supplemented. Bone morphogenetic protein-2 (BMP-2) and transforming growth factor-β1 (TGF-β1) levels were determined using the Human BMP-2 and TGF-β1 ELISA Kits (Sigma-Aldrich, USA), respectively, following the manufacturing instructions. The assays were performed in triplicate.

### hFOB cell culture

From the American Type Culture Collection, a well-established human fetal osteoblast (hFOB 1.19) line was bought. The cells were cultured in DMEM/F-12, which was supplemented with 10% fetal bovine serum (FBS) (Gibco, USA) and 1% penicillin solution (Gibco, USA). The culture flask was placed in an incubator (Thermo, USA) in a humidified atmosphere with 5% CO_2_ at the proliferating condition of 34℃. Every other day, the medium was changed. The population of cells from the sixth to tenth passages was used for the following experiments. The cellular experiments were performed with a two-chamber Transwell system (Corning, USA). Each experiment was independently performed three times.

### Cell proliferation

The hFOB was seeded at a density of 50,000 cells/well into the 24-well plate (Corning, USA), which was used as the lower chamber. To this chamber, 700 µl of DMEM/F-12 medium supplemented with 0.2% FBS and 1% penicillin (0.2% working medium) was added. Then, the upper chamber of the Transwell system was placed with 50 mg TDBS, TDBS-E, or DO BONE. Cells cultured without any material were used as the control group. The plate was incubated at 34℃ and 5% CO_2_. The 3-(4,5-dimethylthiazol-2-yl)-2,5-diphenyltetrazolium bromide (MTT) measurement was performed on days 2 and 3 following the assay instructions.

### Cell migration

Briefly, hFOB were seeded in the upper chamber of the Transwell (pore size of 0.8 μm) at a density of 20,000 cells/well in 100 µl of DMEM/F-12 supplemented with 0.2% FBS and 1% penicillin. In the lower chamber, 100 mg TDBS, TDBS-E, DO BONE, or none (blank control) was added with 600 µl of the same medium. After 1, 2, and 3 days of incubation at 34℃ and 5% CO_2_, the migrated hFOB at the other side of the upper chamber was fixed with 4% paraformaldehyde (PFA) overnight at 4℃ and then were stained with 0.1% crystal violet for 20 min. The migrated hFOB on the membrane was observed under a microscope. In order to quantify the migrated cells, five random microscopic fields were chosen at a magnification of ×200 for cell counting.

### Cell differentiation

The hFOB was seeded into 24-well plates at a density of 75,000 cells/well with 700 µl of the complete medium made up of DMEM/F-12 containing 10% FBS and 1% penicillin. Cells were co-cultured at 39℃ and 5% CO_2_ condition by placing 50 mg of TDBS, TDBS-E, DO BONE, or none in the upper chamber of the Transwell system, respectively. The media were replaced every other day. Alkaline phosphatase activity (ALP) was examined on days 3, 5, and 7 according to the manufacturer’s instructions for the ALP kit (Fluorometric) (Abcam, UK). Fluorometric detection was monitored at Ex/Em = 360/440 nm with the spectrophotometer. ALP activity was normalized by the total protein concentration, which was determined with a BCA protein assay kit (Novagen, USA) and detected by the spectrophotometer; absorbance at 562 nm. The formation of the bone nodule was investigated by alizarin red staining (ARS) on days 10 and 14. The stained Ca deposits were evaluated by digital images captured with Amersham ImageQuant™ (Cytiva, USA) and analyzed by IQTL™ (Cytiva, USA) software.

### Part II: in vivo study

#### Animals

Twenty-four healthy adult male Sprague-Dawley (SD) rats, 8 weeks of age, weighing approximately 200 ± 20 g, were used in this study (provided by the Department of Laboratory Animal, Kunming Medical University, China). The experimental animal protocol was approved by the Ethical Review Committee of Animal Experiments, Kunming Medical University, China (No. kmmu2020241) before the beginning of the experiment and was monitored throughout the experimental process. All methods are reported in accordance with ARRIVE guidelines.

### Surgical procedures

**Fig. 1:**
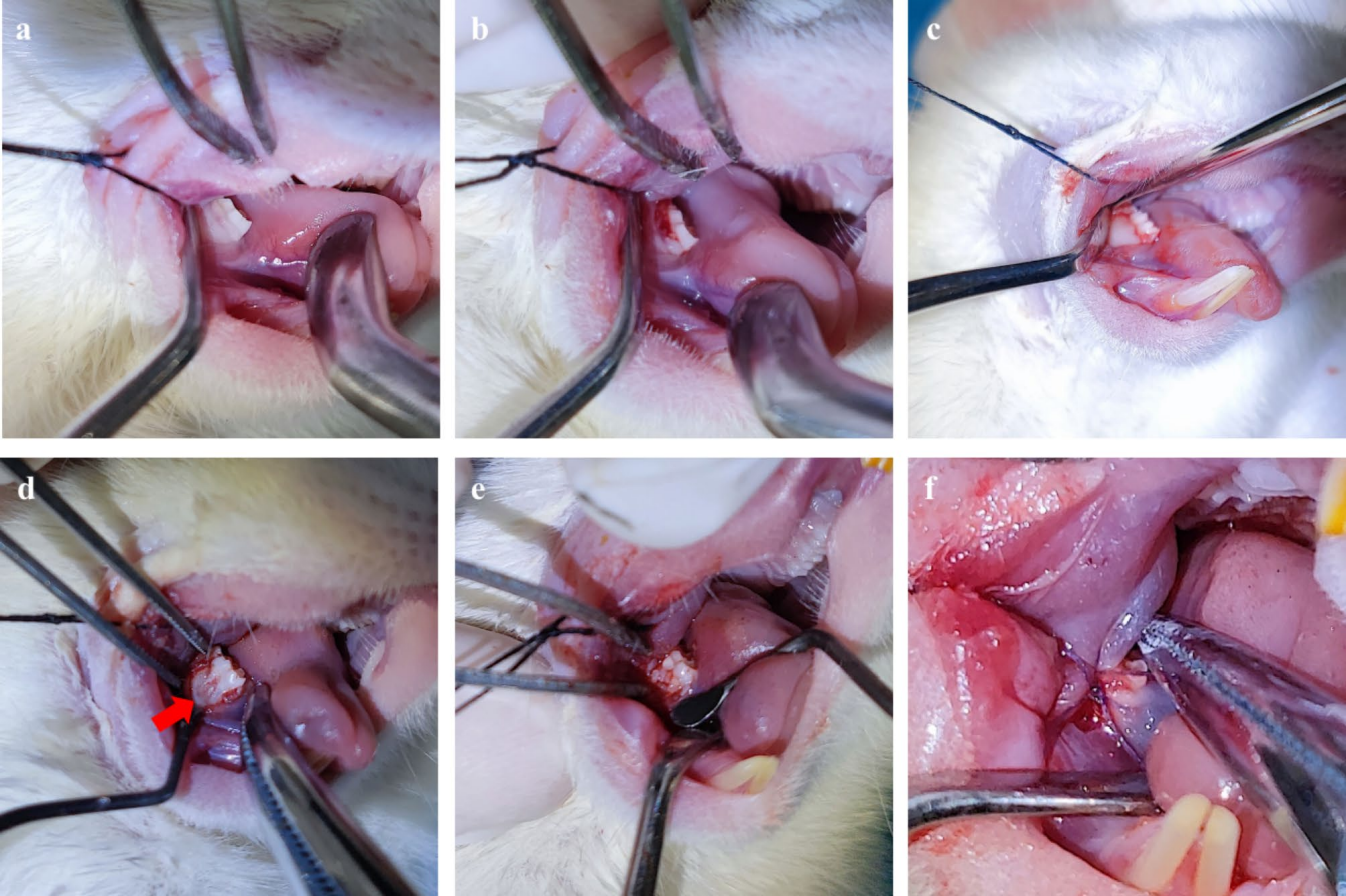
Brief surgical procedures of Grade II furcation in rat periodontal defect model. (**a)** Before incision, (**b)** incision, (**c)** open flap, (**d)** after drill (red arrow: drilling area), (**e**) filling graft materials, (**f)** wound closure.

The surgical procedures were designed based on previous studies [23, 24]. Twenty-four SD rats were randomly distributed to four experimental groups: TDBS, TDBS-E, allograft (BIO-GENE), and blank, with six rats in each group (n = 6). Surgery was performed using general anesthesia with an intraperitoneal injection of 3% pentobarbital sodium at a dosage of 40 mg/kg. Under aseptic conditions, the gingival flap of the buccal side was elevated at the right mandibular first molar to expose the tooth and alveolar bone (Fig. 1a, b, and c). A periodontal defect mimicking Grade II furcation involvements was created on the right mandibular first molars with a high-speed ball drill. The periodontal probe was used to monitor and confirm the size of the defect (width × length × depth; 2 × 2 × 1 mm), as shown in Fig. 1d. After that, scaling procedures were performed to carefully remove the residual periodontal ligament, alveolar bone, and cementum. A sterile saline rinse was continuously applied to the surgical site. Immediately afterward, different groups of bone graft materials were used to fill the furcation defects (Fig. 1e), and defects without any materials were selected as the control. Subsequently, gingival flaps were repositioned and sutured (Fig. 1f). The body weight of all rats was monitored and measured before the surgery and sacrificed to indicate the general health status of the rats during the postoperative period.

### Micro-computed tomography (Micro-CT) analysis

After 8 weeks, the animals were euthanized by cervical dislocation without general anesthesia by trained personnel. Samples containing surgical defect areas were harvested and fixed with 4% PFA for further three-dimensional structural investigation by NEMO™ Micro-CT (PINGSENG Healthcare Inc, China). Avatar 1.5.0 software (PINGSENG Healthcare Inc, China) was used to analyze the data, including bone volume/tissue volume (BV/TV) ratio, bone surface/tissue volume (BS/TV) ratio, trabecular thickness (Th) and trabecular number (N).

### Histological analysis

After radiological analysis, mandibular blocks were decalcified in the Rapid Cal Immuno™ (StatLab, USA) for two weeks. This was followed by dehydration, paraffin embedding, and sectioning. Slices of histological samples, 4 μm in thickness, were produced and stained with hematoxylin-eosin (H&E) for microscopic observation.

### Statistical analysis

Statistical Product and Service Solutions (SPSS) 28.0 (IBM Corp NY, USA) was applied for this experiment. The power analysis has been conducted to obtain the number of animals used in experiments. Statistical analysis confirmed that the above results were a normal distribution with homogeneous variance. All results were parametric and performed by one-way analysis of variance and least significant difference for comparison. The level of statistical significance was set at *p* < 0.05.

## Results

### Dissolution of calcium and phosphate ions from TDBS

**Fig. 2:**
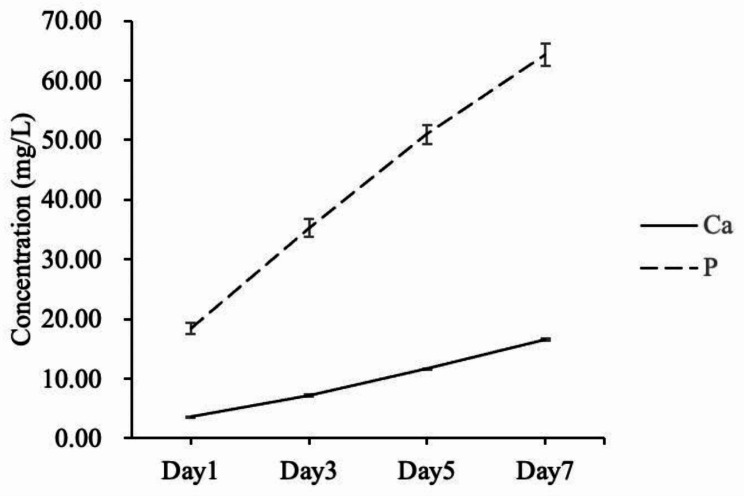
Cumulative values of Ca and P ions dissolution of TDBS from day 1 to 7.

The cumulative value of Ca ion obtained from TDBS was consistently and slightly increased at each time point. Particularly, mean values of 3.58 ± 0.07 mg/L, 7.22 ± 0.13 mg/L, 11.68 ± 0.09 mg/L, and 16.55 ± 0.18 mg/L were recorded on days 1, 3, 5, and 7, respectively (Fig. 2). Notably, the highest Ca dissolution from TDBS was observed on day 7. Simultaneously, the cumulative mean values of P from TDBS measured on the same day exhibited an increasing trend, with values of 18.45 ± 0.99 mg/L, 35.23 ± 1.47 mg/L, 50.96 ± 1.55 mg/L, and 64.28 ± 1.85 mg/L, respectively (Fig. 2).

### The quantification of the released growth factors from TDBS, TDBS-E, and allograft

**Fig. 3:**
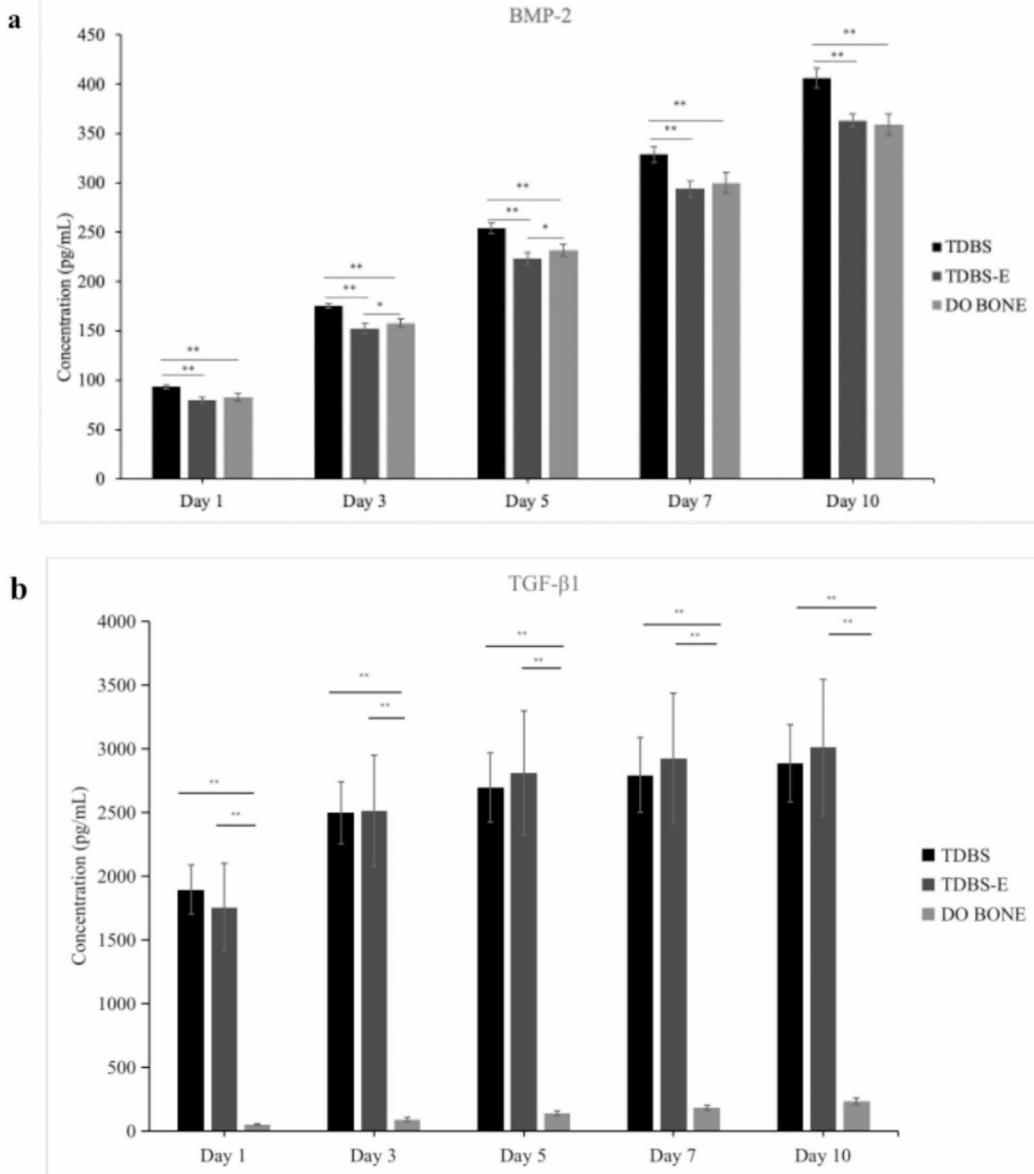
The cumulative value of BMP-2 (**a**) and TGF-β1 (**b**) released from TDBS, TDBS-E, and allograft (DO BONE). *Indicates the level of significance *p* < 0.05; **indicates the level of significance *p* < 0.01

The results of the study indicated that BMP-2 protein was released from TDBS (93.32 ± 2.20 pg/mL), TDBS-E (79.78 ± 3.10 pg/mL), and DO BONE (82.80 ± 3.63 pg/mL) on day 1 and gradually increased until the day 10. The amount of BMP-2 released from TDBS was significantly higher than that released from TDBS-E and DO BONE at every time point (*p* < 0.05), with levels measured at day 10 of 406.14 ± 10.03 pg/mL, 363.33 ± 6.91 pg/mL, and 359.22 ± 11.02 pg/mL, respectively (Fig. 3a). Moreover, on days 3 and 5, the release of BMP-2 from DO BONE (157.98 ± 4.33 pg/mL and 231.71 ± 6.09 pg/mL) was also slightly higher than that from TDBS-E (152.30 ± 5.00 pg/mL and 223.37 ± 6.28 pg/mL).

The TGF-β1 protein levels in the TDBS (1,894.39 ± 193.03 pg/mL) and TDBS-E (1,754.78 ± 345.33 pg/mL) groups were substantially higher than those in the DO BONE group (50.87 ± 8.11 pg/mL) from day 1 onwards, as shown in Fig. 3b. The cumulative values of TDBS (2,887.89 ± 304.90 pg/mL) and TDBS-E (3,010.58 ± 532.18 pg/mL) increased over 10 days, while the TGF-β1 protein level in the DO BONE group remained relatively low, with a cumulative value of only 233.92 ± 24.29 pg/mL until day 10.

### Effect of TDBS, TDBS-E, and allograft on the proliferation and differentiation of hFOB

**Fig. 4:**
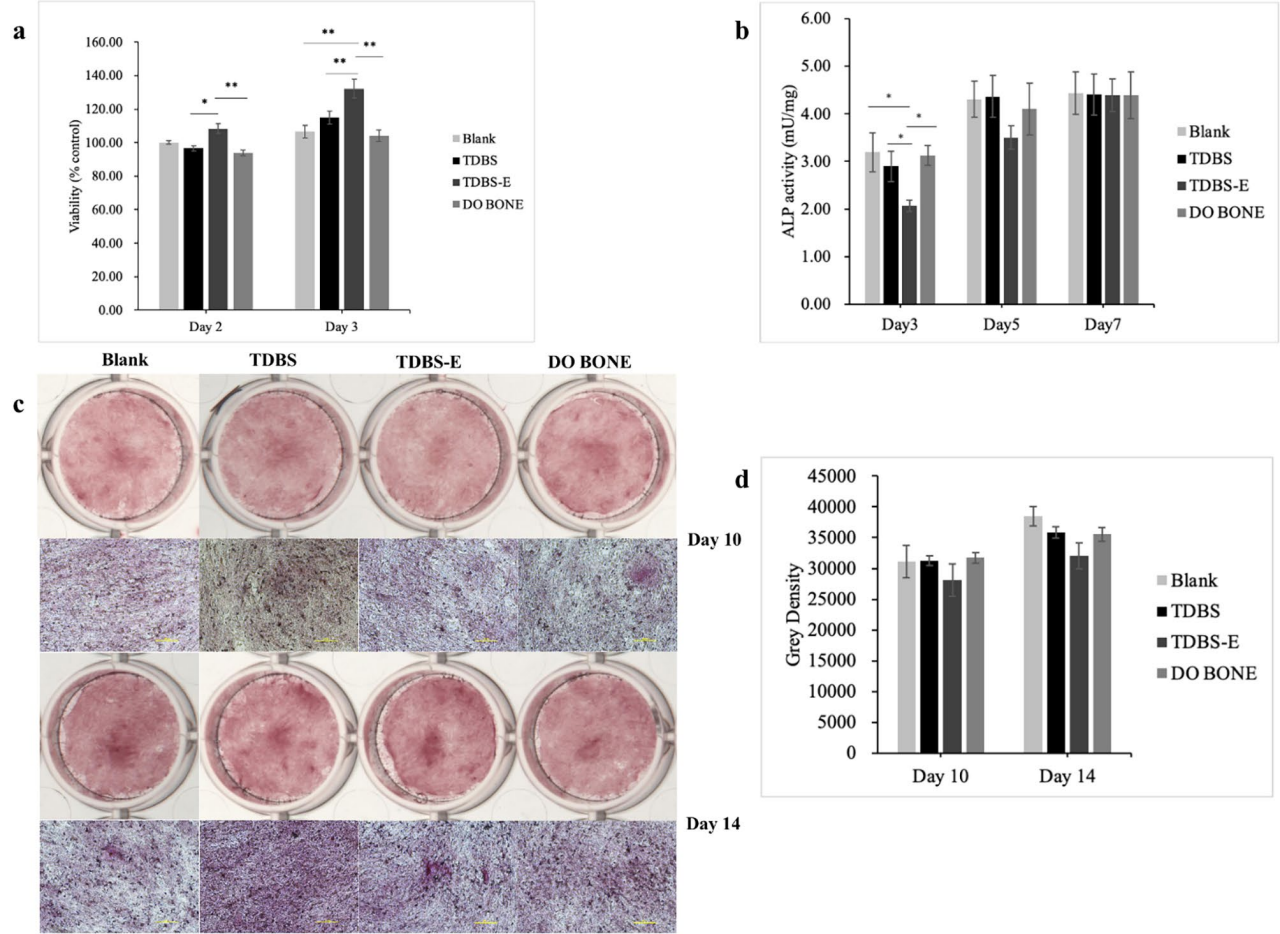
hFOB proliferation (**a**), osteoblastic ALP activity (**b**), mineral nodule formation (**c**), and analysis of alizarin red intensity (**d**) in co-culture with TDBS, TDBS-E, and DO BONE. Scale bar = 100 μm; * *p* < 0.05; ** *p* < 0.01

Under the culture conditions between osteoblasts and different grafting materials, it was observed that the cells were able to proliferate to varying degrees. Even in the blank group, the cell viability was maintained to a small amount of proliferation until at least the third day. The TDBS-E group exhibited the highest ability to induce cell proliferation, as shown in Fig. 4a. At day 3, the cell viability of the TDBS-E group was 132.23 ± 5.61%, which was significantly higher than the blank (106.68 ± 3.73%), TDBS (114.95 ± 3.87%), and DO BONE (104.25 ± 3.47%).

The ALP activity normalized by the total protein concentration showed that there was no difference in ALP/total protein between all groups on days 5 and 7 (Fig. 4b). Interestingly, on day 3, the ALP activity in the TDBS-E group (2.06 ± 0.12 mU/mL) was lower than the TDBS (2.90 ± 0.32 mU/mL), DO BONE (3.12 ± 0.21 mU/mL) groups and the blank group (3.19 ± 0.41 mU/mL). In addition, we did not observe any difference between mineral nodule formation of hFOB in all groups (Fig. 4c and d).

**Fig. 5:**
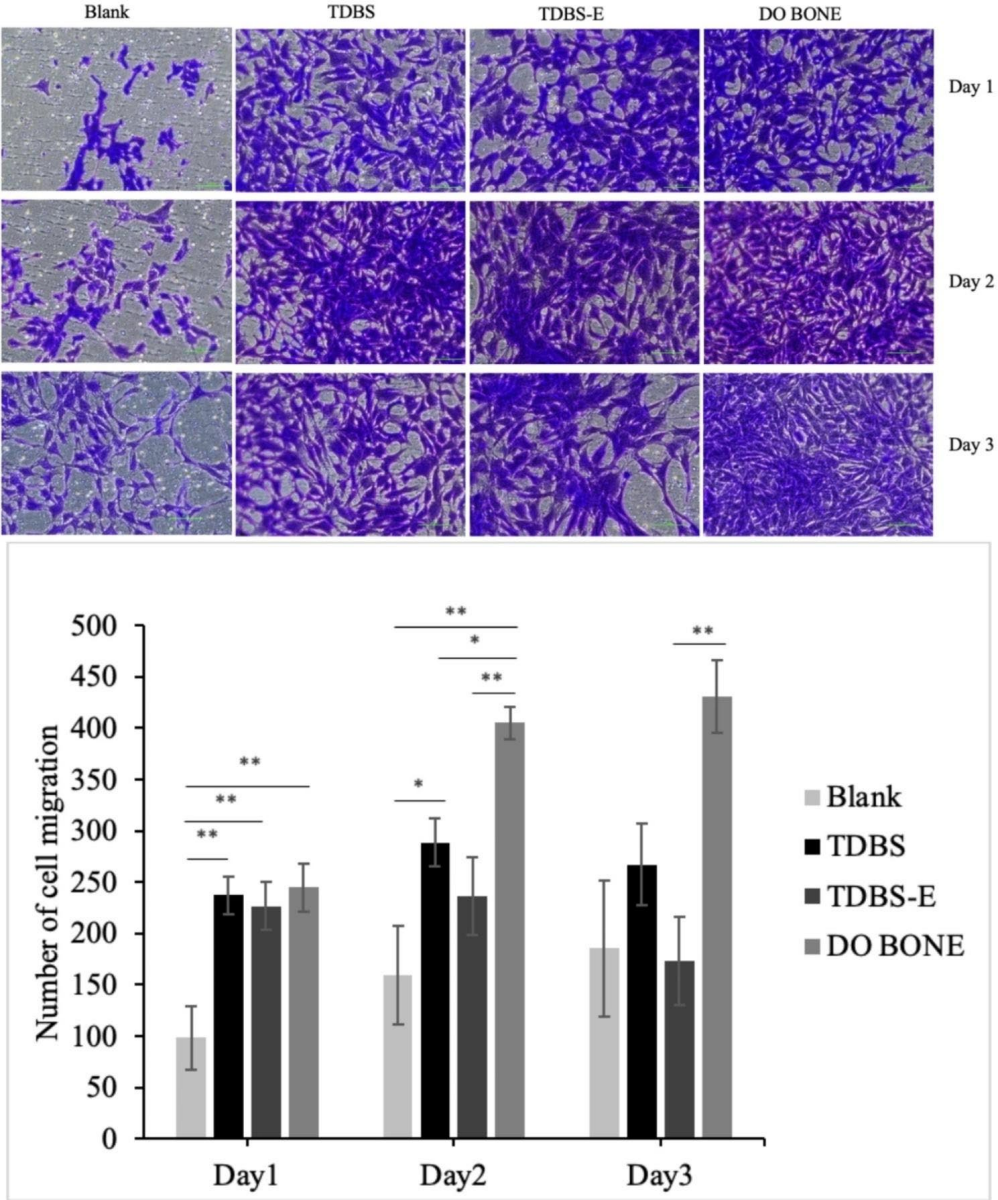
Microscopic observation of the migration and the number of hFOB migrations in co-culture with TDBS, TDBS-E, and DO BONE. Scale bar = 50 μm * *p* < 0.05; ** *p* < 0.01

### Effect of TDBS, TDBS-E, and allograft on the migration of hFOB

To investigate the osteoinductivity of TDBS, TDBS-E, and allograft on osteoblast migration, hFOB cells were cultured on the membrane of the upper chamber while the lower chamber was filled with blank, TDBS, TDBS-E, and allograft. The hFOB in the blank group rarely migrated to the bottom of the chamber on the first day (98.14 ± 31.00). In contrast, the TDBS (237.00 ± 18.87), TDBS-E (226.86 ± 23.33), and DO BONE (245.14 ± 23.38) groups showed a significantly higher number of migrated cells compared to the blank group (Fig. 5). Furthermore, there was a significant increase in the cell migration in the DO BONE group starting from day 2 (405.06 ± 15.47), which was higher than the blank (159.54 ± 48.31), TDBS (288.86 ± 23.77) and TDBS-E (236.80 ± 37.74) groups.

### The effects of TDBS, TDBS-E, and allograft on new bone regeneration in rat periodontal defect model

**Fig. 6:**
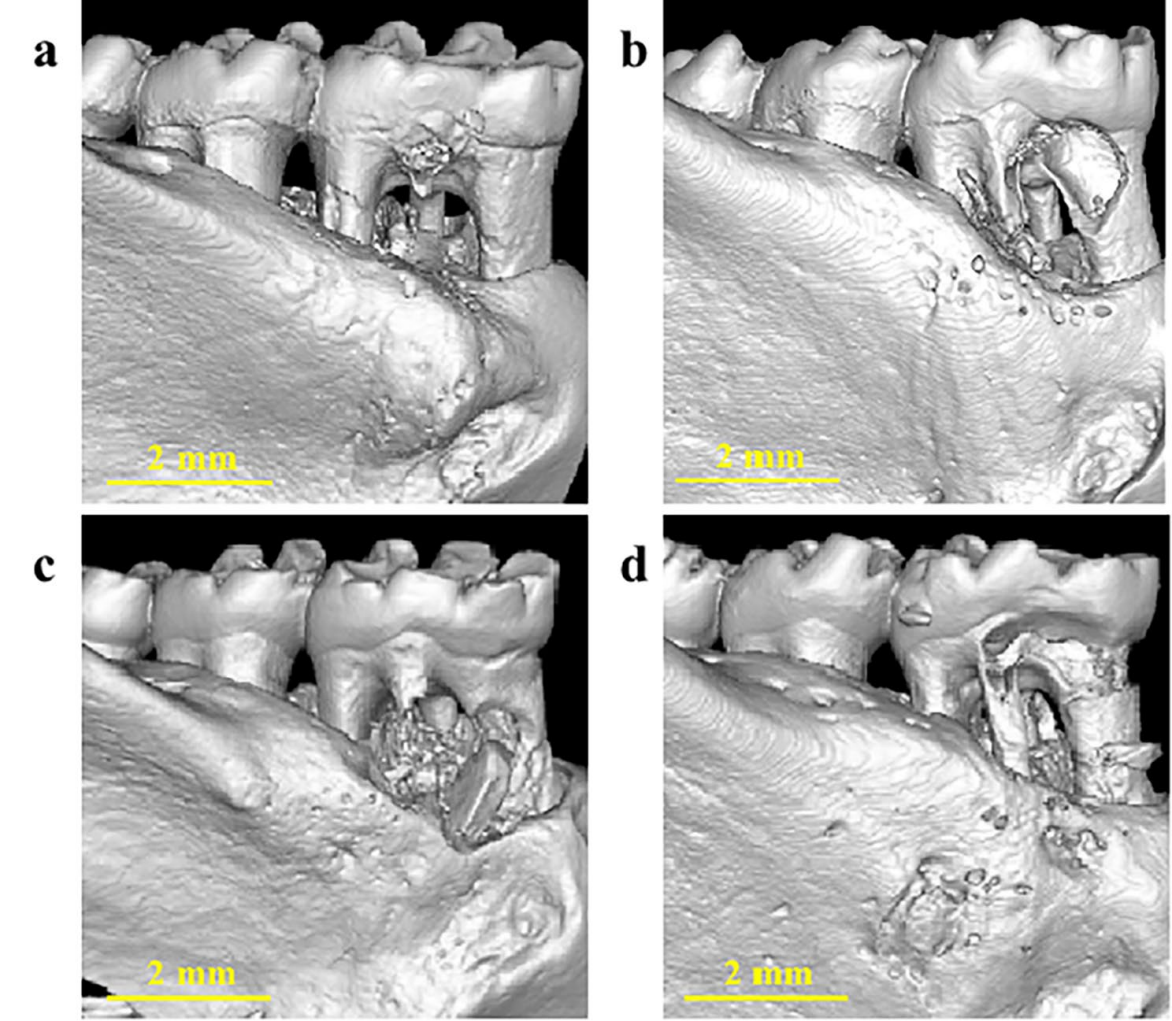
Representative Micro-CT reconstruction images of blank (**a**), TDBS (**b**), TDBS-E (**c**), and allograft (BIO-GENE) (**d**) groups. Scale bar = 2 μm

**Fig. 7:**
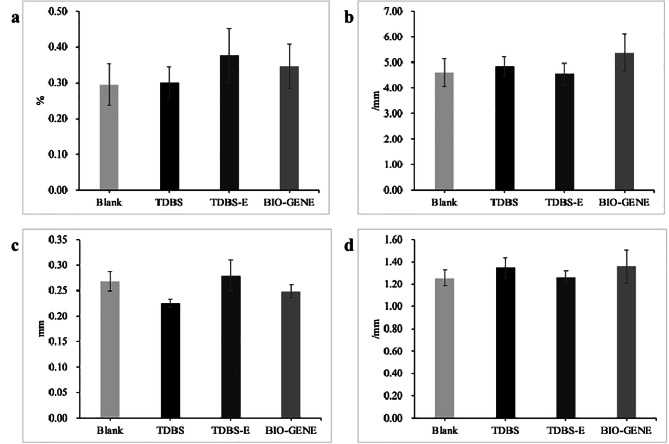
BV/TV (**a**), BS/TV (**b**), Th (**c**), and N (**d**) of periodontal furcation defects in rats.

To further investigate the osteoinductive and biological effects of TDBS under the physiological response of a periodontal defect model, Micro-CT analysis was performed after TDBS, TDBS-E, and allograft were implanted for 8 weeks. The analysis of the three-dimensional (3D) image demonstrated effectively enhanced bone regeneration at the furcation defect in the rat periodontal model eight weeks postoperatively, as shown in Fig. 6. The BV/TV in the TDBS-E (0.38 ± 0.08%) and allograft (BIO-GENE) (0.35 ± 0.06%) groups demonstrated relatively higher volume compared to the blank (0.30 ± 0.06%) and TDBS (0.30 ± 0.04%) groups as shown in Fig. 7a, but the statistical difference between the groups was not significant. The BS/TV of the allograft group showed a trend of slightly higher volume (5.38 ± 0.73 /mm) than other groups (Fig. 7b). The Th and N of bone trabeculae were similar in all four groups (Fig. 7c and d).

**Fig. 8:**
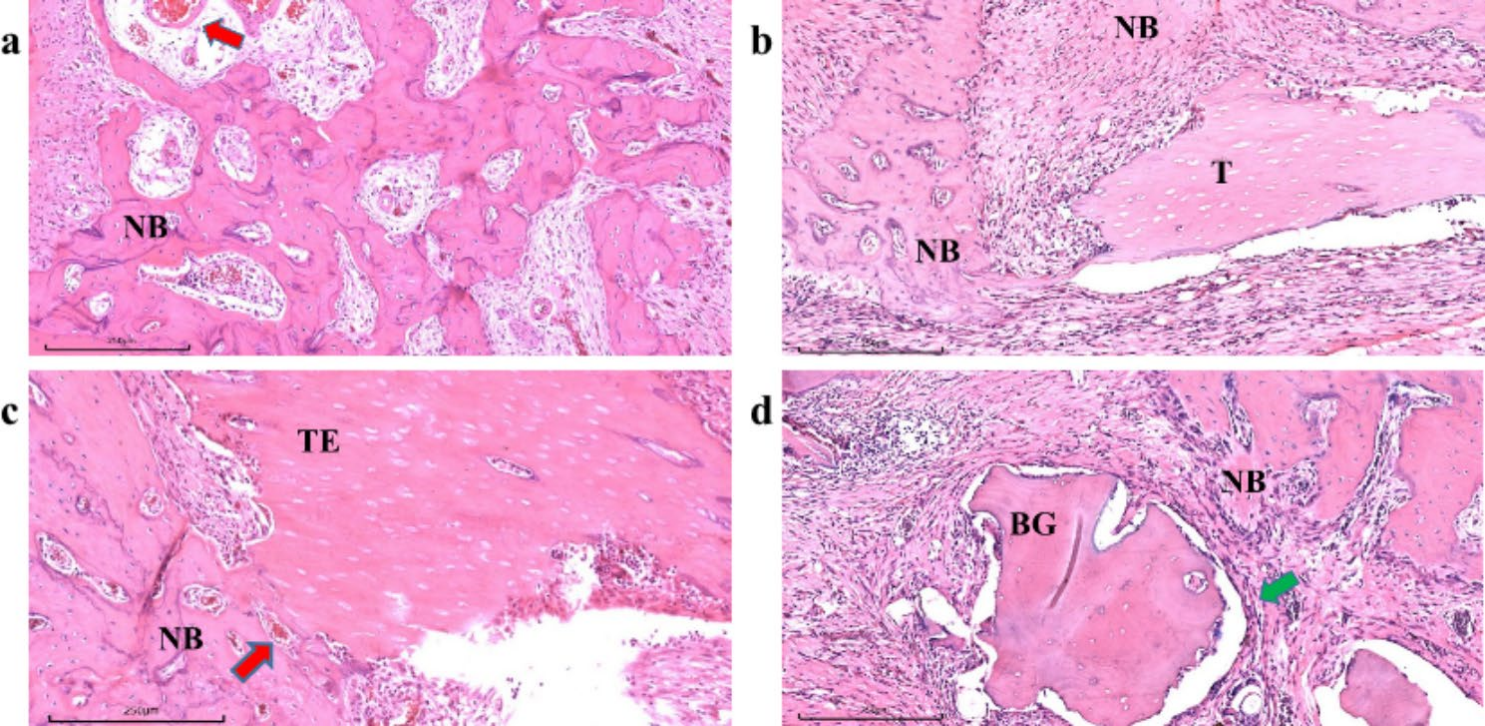
Representative histological finding of blank (**a**), TDBS (**b**), TDBS-E (**c**), and allograft (**d**) groups (NB: new bone; T: TDBS; TE: TDBS-E; BG: BIO-GENE; red arrow: blood vessels invasion; green arrow: fibrous tissue; scale bar = 250 μm).

**Fig. 9:**
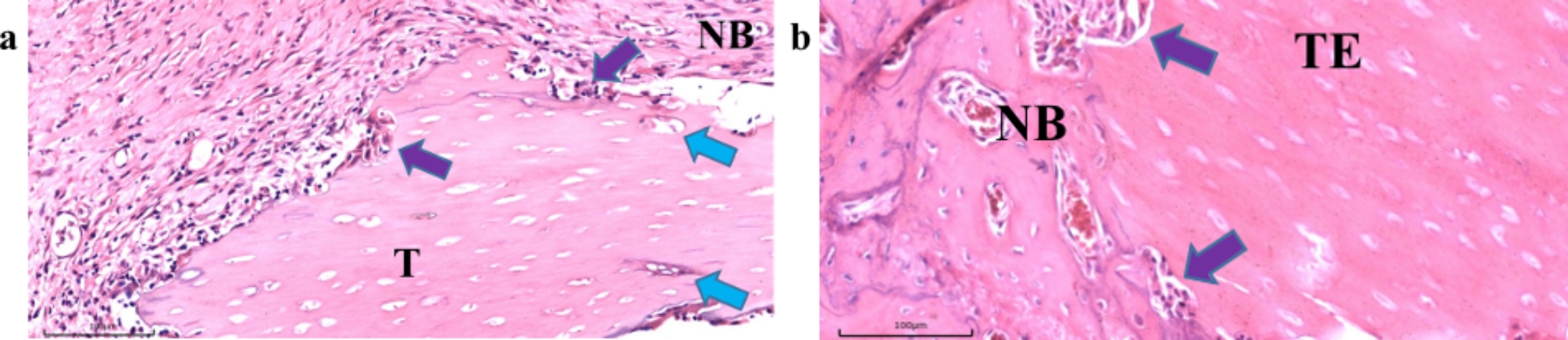
The magnified histological finding of TDBS (**a**) and TDBS-E (**b**) groups (NB: new bone; T: TDBS; TE: TDBS-E; purple arrow: marginal bone resorption; blue arrow: cells migrated into the dentin tubules; scale bar = 100 μm).

At eight weeks postoperatively, new bone formation was observed in all four groups to a varying degree. In the material groups, new bone formation was observed around the margins of the graft (Fig. 8). The blank group showed regeneration of cancellous bone, displaying an irregular arrangement with loose connective tissue interstices and new blood vessels invasion between them (red arrow in Fig. 8a). Moreover, in the TDBS and TDBS-E groups, it was noted that a small number of cells had grown into the dentin tubules of the implanted material. The edges of the material also began to resorb and tightly attached to the newly formed bone and connective tissues (Fig. 9). However, in the allograft group, a layer of fibrous tissue (indicated by a green arrow in Fig. 8d) wrapped around the material was observed, along with an increase of fibroblasts embedded in the edges of the material. The presence of inflammatory infiltration surrounding the graft was also observed in the TDBS and allograft groups (Fig. 8b and d).

## Discussion

The extracted teeth, which are classified as biomedical waste and discarded, as bone substitute materials in clinical trials and animal studies have demonstrated advantages in terms of easy accessibility, and specific bone regeneration-promoting properties are highly favored by clinicians and researchers [11, 12]. Previous studies [25] have shown that autogenous dental tissue used as bone graft material can be divided into whole tooth and dentin. Dentin can be processed into completely undemineralized dentin, partially demineralized dentin matrix, and completely demineralized dentin matrix, depending on the degree of demineralization. Demineralization aids in promoting the release of proteins like growth factors, and therefore, DDM has shown high biocompatibility with osteoconductivity and osteoinductivity in several studies. However, the preparation of DDM is time-consuming, as the demineralization process generally requires strong acidic conditions as well as a proper laboratory setting, which makes it impractical in clinical settings. Binderman et al. [15] first proposed the use of TDBS in 2014 and designed an SDG machine for processing the whole extracted tooth as bone grafting materials for immediate applications such as socket preservation or dental implant surgery. Recent clinical case reports and systematic reviews have shown that undemineralized autogenous tooth grafts can be considered acceptable biomaterials for different bone defects, with favorable results [17, 26, 27].

Calcium can induce bone tissue regeneration in bone growth precursor cells, stimulate the osteosynthetic pathway in osteoblasts, and prolong the lifespan of osteoblasts [28]. Phosphorus, mainly in the form of phosphate, regulated the growth and differentiation of osteoblasts and osteoclast lineages and was also reported to enhance the expression of BMPs [29]. Our calcium and phosphate dissolution assay revealed that both Ca and P were increasingly released from TDBS over time, similar to previous reports showing that the solubility of Ca in TDBS increased gradually from day 3 to day 14, similar to mandibular ramus bone, autologous block bone, and allografts [21].

The determination of scaffold material for cell growth and tissue regeneration both in vitro and in vivo is a crucial component of bone tissue regeneration engineering. In this study, hFOB co-cultured with demineralized TDBS exhibited higher proliferation rates on days 2 and 3, particularly by day 3, with the demineralized TDBS group demonstrating a significant advantage over the blank group, TDBS group, and allograft group. This trend was maintained until day 5 in our preliminary study, suggesting the anabolic effect of TDBS-E on osteoblast proliferation. It was reported [14] that there was no difference in cell proliferation on dentin particles, whether demineralized or not. However, for enamel, demineralized enamel increased cell proliferation more than undemineralized enamel. In physicochemical characterization analysis, it was found that demineralized TDBS had a smoother surface after demineralization. Notably, the dentin tubules were wider after demineralization than non-demineralization [13, 14]. Previous studies have shown that human osteosarcoma cell lines (MG-63) on smooth surfaces have the propensity to proliferate, differentiate, and generate local promoting factors such as TGF-β1 and prostaglandin E_2_ [30]. A significant upregulation of TGF-β1 was observed in our results from the TDBS-E group, suggesting that TDBS-E may release a certain amount of osteogenic regulatory factors, such as TGF-β1 and BMP-2, which provided support for cell proliferation to some extent. Therefore, we speculated that the smooth surface and wider dentinal tubules of demineralized TDBS might be more favorable for the release of growth factors to the proliferation of hFOB, with enamel displaying an advantage in cell proliferation after demineralization.

The numbers of osteoblast migrated in all materials were significantly higher than the blank control at the earliest day, indicating that non-demineralized TDBS, demineralized TDBS, and allograft had a strong chemotactic effect on osteoblast migration. The BMP-2 protein was found to be involved in the chemotactic recruitment of osteoblasts throughout the bone formation, as evidenced by dose-dependently correlation with human primary osteoblast migration [31]. This present study demonstrated that all materials continuously released BMP-2 for 10 days, with non-demineralized TDBS being particularly significant. The release of BMP-2 protein peaked around day 21 in the dental pulp stem cells (DPSCs) cultured with TDBS [32], confirming that TDBS has excellent potential as an osteogenic protein carrier substance, especially after interacting with cells. This result may also explain the higher ALP activity shown in TDBS compared to the demineralized TDBS in the early stage. Moreover, TGF-β1 could trigger the synthesis of BMPs in the osteoprogenitor cells to facilitate osteoblast differentiation [33]. As time moved on, all material groups showed similar ALP activity by days 5 and 7 and were able to promote matrix calcification through calcium deposition on days 10 and 14, which may be related to the high amounts of TGF-β1 released from non-demineralized TDBS and demineralized TDBS over 10 days. Yang et al. demonstrated that the DDM particles could sustain the release of TGF-β1 until day 13 [34]. The TDBS as scaffold induced a more fabulous presence of ALP at days 7, 14, and 21, verifying its ability to support faster extracellular matrix mineralization at a later stage [32]. Our results provide convincing evidence that at least two proteins associated with osteogenesis and bone remodeling, including BMP-2 and TGF-β1, could be preserved and released from both non-demineralized TDBS and demineralized TDBS, making them clinically suitable as bone graft materials.

A Grade II furcation bone defect model of the mandibular first molar was created in order to investigate the potential osteogenic role of the TDBS in a periodontal defect. It created an environment favorable for periodontal regeneration, similar to a three-walled bone pocket [35]. The results of BV/TV, BS/TV, Th, and N from all groups effectively enhanced bone regeneration in the furcation area in the periodontal defect model. In comparison, implantation of DDM increased new bone formation in rat calvarial bone defects starting at 2 to 8 weeks [25, 34], and combined cell sheets use increased bone volume in one-wall periodontal intrabony defects of a dog model at 8 weeks, evidencing the early formation of new bone tissue compared to blank group [34]. We speculated that non-demineralized TDBS or demineralized TDBS might have an advantage in inducing new bone formation at about four weeks, while at eight weeks, bone volume might not be entirely superior until the new bone has replaced the graft material. Similar models of periodontal defects in rats support this assumption, where regeneration of periodontal ligament-like tissue and new bone could be observed at weeks 2, 4, and 6 [24, 36]. Furthermore, the osteogenic role of non-demineralized dentin was also confirmed in the study by Farzad et al. [37], in which no difference in the bone-to-implant contact and the new bone filling area was observed between non-demineralized and demineralized dentin block in a variety of EDTA demineralized conditions, suggesting that the degree of demineralization may have less influence on bone regeneration than other factors.

On histological sections, fibrous connective tissue was observed to encapsulate some of the margins of non-demineralized TDBS, demineralized TDBS, and allogeneic bone material, as dentin grafts were mobile during the osteogenic phase of bone healing [38]. This is strongly related to our choice of the surgical area for the first molar in rats, which was affected by their masticatory and muscular movement, simulating the implantation more realistically in an actual patient’s mouth. We did not observe the obvious formation of periodontal ligament-like tissue along the root in furcation defect areas, which may be associated with the complexity of the anatomical structures in the furcation area and insufficient recruitment of periodontal ligament cells. The observation of a certain degree of inflammatory infiltration around the bone graft material is likely associated with the fact that the TDBS or allograft implanted into the rats originates from human teeth and bones, both of which are xenogeneic to rats, potentially causing an immune response. Additionally, the surgery itself could lead to tissue damage and an inflammatory response in the surrounding tissues, which is typically a part of the normal healing process. Periodontal tissue engineering faces challenges due to the diversity and uncontrollable regenerative environment of periodontal pockets and bone defects. Scaffold materials need to be multi-phase in terms of providing both basic osteoconductivity and an osteoinductive microenvironment. This study demonstrated the potential of TDBS as a scaffold for periodontal tissue engineering, with the ability to induce bone formation comparable to commercial bone grafting material without requiring complex demineralization procedures and lengthy processing times. However, the additional osteogenic-related genes and proteins (i.e., Runx2, collagen type I, VEGF, other BMPs, etc.) should be further explored. Furthermore, it would be more advantageous to conduct histological analysis with an increased number of observation time points in further animal studies. Although we successfully induced a periodontal bone defect model, there was an impossibility of self-repair of alveolar bone loss related to long-term chronic inflammation in human periodontal furcation involvement, which could be a difference in the animal model. The further study may also include models that mimic alveolar bone loss in a variety of furcation defects under the oral micro-ecosystem to provide more knowledge about the optimal periodontal regenerative treatment.

## Conclusions

The TDBS from extracted teeth is obtained non-invasively and has low infection and rejection risks. This present study revealed that TDBS can act as a carrier for growth factors like BMP-2 and TGF-β1. The TDBS also induced proliferation, migration, and differentiation in co-cultured osteoblasts and promoted bone and tissue regeneration in a periodontal furcation bone defect model. Overall, TDBS exhibited a competitive and excellent alternative to allografts, showing comparable osteoconductive and osteoinductive properties. Therefore, chairside-prepared TDBS was suggested as an economical and biocompatible material for periodontal bone regeneration and other dental surgery.

### Electronic supplementary material

Below is the link to the electronic supplementary material.

Supplementary Material 1

## Data Availability

The datasets used and/or analyzed during the current study are available from the corresponding author on reasonable request.

## Abbreviations

TDBS: Tooth-derived bone substitute
EDTA: Ethylene diamine tetraacetic acid
TDBS-E: TDBS treated with EDTA
Ca: Calcium
P: Phosphate
BMP-2: Bone morphogenetic protein-2
TGF-β1: Transforming growth factor-β1
ELISA: Enzyme-linked immunosorbent assay
MTT: 3-(4,5-dimethylthiazol-2-yl)-2,5-diphenyltetrazolium bromide
ALP: Alkaline phosphatase activity
ARS: Alizarin red staining
Micro-CT: Micro-computed tomography
BMPs: Bone morphogenic proteins
TGF-β: Transforming growth factor-β
RUNX2: runt-related transcription factor 2
DDM: Demineralized dentin matrix
hFOB: Human fetal osteoblast
SDG: Smart dentin grinder
DI: Deionized
DMEM/F-12: Dulbecco’s modified eagle medium/nutrient mixture F-12
FBS: Fetal bovine serum
PFA: Paraformaldehyde
SD: Sprague-Dawley
BV/TV: Bone volume/tissue volume
BS/TV: Bone surface/tissue volume
Th: Thickness
N: Number
H&E: Hematoxylin-eosin
SPSS: Statistical Product and Service Solutions
3D: three-dimensional
MG-63: Human osteosarcoma cell lines
DPSCs: Dental pulp stem cells
