# Supplementary material for: Bone regeneration property of tooth-derived bone substitute prepared chairside for periodontal bone defects: an experimental study
Source: BMC Oral Health. 2023 Nov 14;23:863. doi: 10.1186/s12903-023-03582-y (PMC10647160; doi:10.1186/s12903-023-03582-y)
Supplement: Supplementary file 1 — Supplementary Material 1 [file 12903_2023_3582_MOESM1_ESM.docx]

Supplemental figure and tables

**
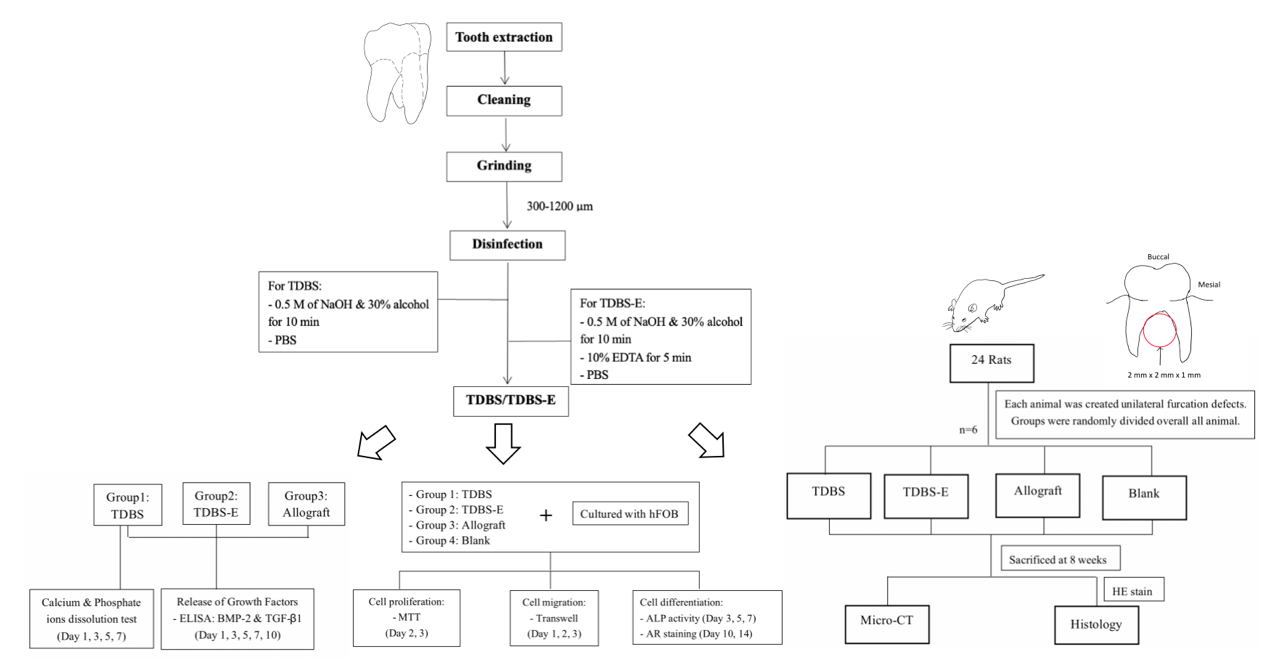
**

**Figure S1 The design of the in vitro and in vivo experiments and the steps for teeth preparation.**

**Table S1 Four materials used in this study**

| **Name** | **Source** | **Bone graft type** | **Composition** | **Size** |
| --- | --- | --- | --- | --- |
| TDBS | Teeth (Human) | Autogenous tooth particles | Enamel, dentin, and cementum | 300-1200 µm |
| TDBS-E | Teeth (Human) | Demineralized autogenous tooth particles | Demineralized Enamel, dentin, and cementum | 300-1200 µm |
| DO BONE | Bone (Human) | Allograft | Cancellous and cortical bone | 400-710 µm |
| BIO-GENE | Bone (Human) | Allograft | Cancellous and cortical bone | 300-1200 µm |

**Table S2 Inclusion and exclusion criteria of teeth collection**

| **Inclusion criteria** | - Patients aged 13-50 years - Non-smokers - Non-alcoholics - Teeth extracted due to impaction or for orthodontic purposes |
| --- | --- |
| **Exclusion criteria** | - Patients with infectious diseases, such as hepatitis B, hepatitis C, or human immunodeficiency virus - Teeth with extensive caries - Previously undergone dental treatments: including fillings, root canal treatment, restorations, or prosthetic crowns - Tooth malformation or congenital dental anomalies, such as fluorosis, enamel hypoplasia, amelogenesis imperfecta, dentinogenesis imperfecta, and dentin dysplasia |
